# Reshaping [^99m^Tc]Tc-DT11 to DT14D Tagged with Trivalent Radiometals for NTS_1_R-Positive Cancer Theranostics

**DOI:** 10.3390/pharmaceutics17030310

**Published:** 2025-02-28

**Authors:** Panagiotis Kanellopoulos, Berthold A. Nock, Eric P. Krenning, Theodosia Maina

**Affiliations:** 1 Molecular Radiopharmacy, INRaSTES, NCSR “Demokritos”, 15341 Athens, Greece; kanelospan@gmail.com (P.K.); nock_berthold.a@hotmail.com (B.A.N.); 2 Cyclotron Rotterdam BV, Erasmus MC, 3015 CE Rotterdam, The Netherlands; erickrenning@gmail.com

**Keywords:** cancer radiotheranostics, neurotensin subtype 1 receptor, trivalent radiometal, DOTA-peptide conjugate, metabolic stability, neprilysin

## Abstract

**Background/Objectives**: Radiotheranostics of neurotensin subtype 1 receptor (NTS_1_R)-expressing tumors, like pancreatic, gastrointestinal, or prostate cancer, has attracted considerable attention in recent years. Still, the fast degradation of neurotensin (NT)-based radioligands, by angiotensin-converting enzyme (ACE), neprilysin (NEP), and other proteases, has considerably compromised their efficacy. The recently introduced [^99m^Tc]Tc-DT11 (DT11, N_4_-Lys(MPBA-PEG4)-Arg-Arg-Pro-Tyr-Ile-Leu-OH; N_4_, 6-(carboxy)-1,4,8,11-tetraazaundecane) has displayed promising uptake in NTS_1_R-positive tumors in mice and enhanced resistance to both ACE and NEP by virtue of the lateral MPBA-PEG4 (MPBA, 4-(4-methylphenyl)butyric acid; PEG4, 14-amino-3,6,9,12-tetraoxatetradecan-1-oic acid) chain attached to the ε-NH_2_ of Lys^7^. We were next interested in investigating whether these qualities could be retained in DT14D, likewise modified at Lys^7^ but carrying the universal chelator DOTA (1,4,7,10-tetraazacyclododecane-1,4,7,10-tetraacetic acid) via a (βAla)_3_ spacer at the α-NH_2_ of Lys^7^. This chelator switch enables the labeling of DT14D with a wide range of trivalent radiometals suitable for true theranostic applications, not restricted to the diagnostic imaging of NTS_1_R-positive lesions only by single-photon emission computed tomography (SPECT). **Methods**: DT14D was labeled with Ga-67 (a surrogate for the positron emission tomography radionuclide Ga-68), In-111 (for SPECT), and Lu-177 (applied in radiotherapy). The resulting radioligands were tested in NTS_1_R-expressing pancreatic cancer AsPC-1 cells and mice models. **Results**: [^67^Ga]Ga/[^111^In]In/[^177^Lu]Lu-DT14D displayed high affinity for human NTS_1_R and internalization in AsPC-1 cells. They remained >70% intact 5 min after entering the mice’s circulation, displaying NTS_1_R-specific uptake in AsPC-1 xenografts. **Conclusions**: Suitably side-chain modified NT analogs show enhanced metabolic stability and hence better prospects for radiotheranostic application in NTS_1_R-positive cancer.

## 1. Introduction

Target-specific radiopharmaceuticals have enabled personalized diagnosis and therapy—“radiotheranostics”—of human cancer, inherently harmonizing with the principles of modern precision medicine [1,2,3]. The first clinical success of radiolabeled somatostatin analogs in the treatment of neuroendocrine tumors [4] has fostered research toward other peptide-based analogs targeting alternative biomolecular targets overexpressed in a plethora of human tumors [5]. For example, the native 13 amino acid neurotensin (pyroGlu-Leu-Tyr-Glu-Asn-Lys-Pro-Arg-Arg-Pro-Tyr-Ile-Leu-OH), involved in various physiological functions in the nervous system and in the gut [6], has been shown to exert mitogenic effects in a number of human cancers [7,8,9] via the neurotensin subtype 1-receptor (NTS_1_R). Among the three neurotensin receptors (NTS_1_R, NTS_2_R, and NTS_3_R), the NTS_1_R is definitely the most relevant in oncology [6], owing to its high-density expression in various human cancers [10,11,12], including colorectal cancer [7,13,14], pancreatic ductal adenocarcinoma [15,16,17,18], lung cancer [19], Ewing’s sarcoma [20], and other malignant tumors [21,22,23].

Along these lines, a number of NT analogs have been developed over the years to deliver medically relevant radiometals to NTS_1_R-positive lesions. Most of these analogs are based on the C-terminal hexapeptide of the native sequence, NT(8-13), as the essential fragment retaining full capacity of interaction with the NTS_1_R [24]. Typically, a bifunctional chelator is attached at the N-terminus of NT(8-13) and its derivatives, either directly or via a spacer. For example, an open-chain tetraamine or a bi/tri-dentate chelator in combination with the Tc-tricarbonyl synthon was used for the stable binding of Tc-99m for single-photon emission computed tomography (SPECT) purposes [25,26,27]. Despite this, true radiotheranostic goals can only be reached by means of alternative chelators, such as macrocyclics [28,29,30,31], able to stably bind radiometals applied as well in positron emission tomography (PET) imaging (e.g., Ga-68, Cu-64) [32,33,34] or in radiotherapy (particle emitters, such as Lu-177, or Ac-225) [34,35]. Thus far, clinical translation outcomes in this research area have been rather disappointing, a fact attributed, at least in part, to the poor metabolic stability of NT and its analogs [36,37,38].

Indeed, a number of proteases have been reported to participate in the rapid catabolism of NT in the biological milieu [39,40,41,42]. It is reasonable to assume that, among them, the ones directly coming in contact with the circulating NT radioligand on its rapid ride to the target actually have the opportunity to exert their proteolytic action, eventually compromising tumor targeting, whereas intracellular/compartmentalized proteases are expected to have little if any effect [43,44]. Accordingly, neutral endopeptidase (or neprilysin, NEP; fast cleaving the Pro^10^-Tyr^11^ and the Tyr^11^-Ile^12^ bonds) and angiotensin-converting enzyme (ACE; rapidly hydrolyzing the Tyr^11^-Ile^12^ bond) are the two key proteases expected to fast degrade NT-based radioligands entering the blood-stream in the narrow time-window they need to reach tumor site(s) [45,46,47,48]. Interestingly, during our studies on NTS_1_R-directed radioligands, we were able to demonstrate partial or synergetic improvements in the uptake in implanted tumors in mice by means of single or combined NEP/ACE inhibitors [44,49]. Most importantly, we were able to correlate these findings with marked stability improvements verified in peripheral mice blood at 5 min post-injection (pi) when specific NEP or/and ACE inhibitors were administered, such as the potent NEP inhibitors phosphoramidon (PA) [50,51] or sacubitrilat (in vivo released from the sacubitril precursor present in the registered drug Entresto^®^) [52,53,54] and the clinically used ACE inhibitor lisinopril [48].

**Figure 1:**
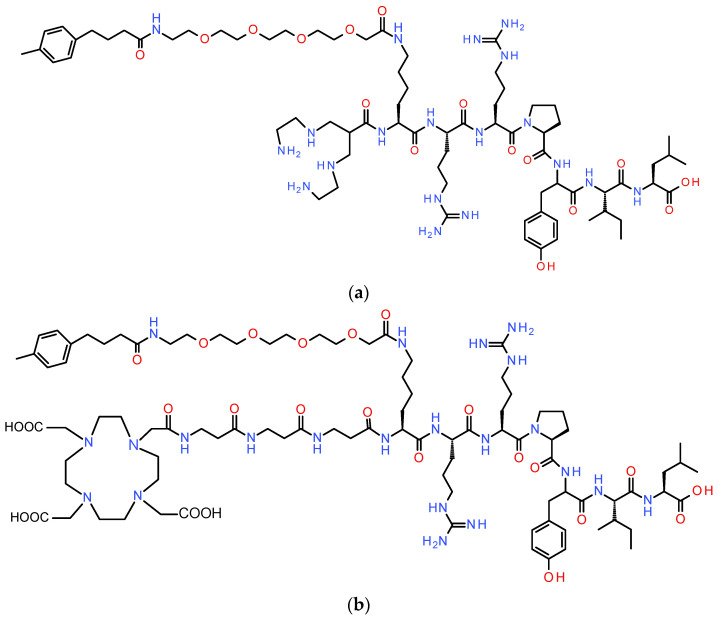
Chemical structures of (**a**) parental DT11 (DT11, N_4_-Lys(MPBA-PEG4)-Arg-Arg-Pro-Tyr-Ile-Leu-OH; N_4_, 6-(carboxy)-1,4,8,11-tetraazaundecane; MPBA, 4-(4-methylphenyl)butyric acid; PEG4, 14-amino-3,6,9,12-tetraoxatetradecan-1-oic acid) suitable for labeling with Tc-99m and (**b**) reshaped to DT14D (DOTA-βAla-βAla-βAla-Lys(MPBA-PEG4)^7^-Arg-Arg-Pro-Tyr-Ile-Leu-OH; DOTA, 1,4,7,10-tetraazacyclododecane-1,4,7,10-tetraacetic acid), thereby enabling labeling with theranostic trivalent radiometals.

Aiming to minimize or circumvent the need to administer single or combined protease inhibitor regimens, which would complicate procedures and protocol approvals for clinical translation, we have attempted to stabilize [^99m^Tc]DT1 (DT1, N_4_-Gly-Arg-Arg-Pro-Tyr-Ile-Leu-OH, N_4_=6-(carboxy)-1,4,8,11-tetraazaundecane) and its mimics by introducing lateral chains on the *ε*-amine of Lys^7^ of the N_4_-Lys(R^x^)-Arg-Arg-Pro-Tyr-Ile-Leu-OH backbone [55,56]. Among the resulting branched [^99m^Tc]DT1 mimics that were screened for their targeting potential in suitable NTS_1_R-positive cell and mice models, [^99m^Tc]DT11 (R^x^, MPBA-PEG4, MPBA, 4-(4-methylphenyl)butyric acid and PEG4, 14-amino-3,6,9,12-tetraoxatetradecan-1-oic acid; Figure 1) appeared to combine improved in vivo stability with good tumor uptake and pharmacokinetics in mice [55,56]. In fact, treatment of mice with Entresto^®^ alone led to higher stabilization in the bloodstream without the need for treatment of mice with Lis or another ACE inhibitor [56].

Encouraged by these findings, we next decided to “reshape” [^99m^Tc]DT11 to a radiotheranostic agent, able to accommodate radiometals for application not only for SPECT imaging (e.g., In-111) but also for PET (e.g., Ga-68) and radionuclide therapy (e.g., the beta emitter Lu-177). For this purpose, we replaced the N_4_ with the universal chelator DOTA (1,4,7,10-tetraazacyclododecane-1,4,7,10-tetraacetic acid) [31] attached via a (βAla)_3_ linker to the *a*-amine of Lys^7^ yielding DT14D (Figure 1). The effect of these modifications on the performance of DT14D labeled with Ga-67 (as a surrogate of the short-lived Ga-68), In-111, and Lu-177 was studied in NTS_1_R-expressing cells and mice models. The suitability of DT14D as a radiotheranostic tool for NTS_1_R-expressing cancer was assessed in direct comparison with the parent [^99m^Tc]DT11, carrying the monocationic *trans*-[^99m^Tc][Tc(O)_2_(N_4_)]^+^-chelate at the N-terminus.

## 2. Materials and Methods

### 2.1. Chemicals, Radionuclides, and Radioligands

#### 2.1.1. Peptides, Peptidase Inhibitors, and Radionuclides

Neurotensin was acquired from Bachem (Bubendorf, Switzerland). The novel peptide-conjugate DT14D was supplied by PiChem Forschungs- und Entwicklungs GmbH (Raaba-Grambach, Austria). The formation of the desired product was confirmed through matrix-assisted laser desorption/ionization-time of flight (MALDI-TOF) mass spectrometry (MS) and high-performance liquid chromatography (HPLC) analysis verified a purity of over 95% (Table S1, Supplementary Materials). The structures of DT14D and its parent compound DT11 are illustrated in Figure 1 [56]. Entresto^®^ pills (200 mg corresponding to 24 mg/26 mg sacubitril/valsartan per pill; Novartis AG, Basel, Switzerland) were obtained from a local pharmacy as a source of the potent and highly specific NEP-inhibitor sacubitrilat [53,54]. The pills were crushed to a fine powder in a mortar and divided into equal portions (12 mg of pill material, equivalent to 1.44 mg of the precursor sacubitril); just before oral gavage to the mice, each portion was suspended in tap water to form a slurry (200 μL) [49,55,56]. Solvents and common chemicals were reagent grade and sourced from various commercial suppliers, except for HPLC solvents, which were of HPLC grade. The trivalent metal-tagged DT14D were prepared using the nitrate salts [^nat^Ga]Ga(NO_3_)_3_, [^nat^In]In(NO_3_)_3_, and [^nat^Lu]Lu(NO_3_)_3_ (Merck SA, Athens, Greece).

For radioiodination, [^125^I]NaI in dilute sodium hydroxide solution (pH 8–11) was purchased from Selidis, A., Bros—Antisel S.A. (Athens, Greece). [^67^Ga]GaCl_3_ (4–5.5 GBq/mL in dilute HC1 solution) was supplied by IDB Holland B.V. (Baarle-Nassau, The Netherlands), whereas [^111^In]InCl_3_ (400–600 MBq/mL in 0.05 mM HCl) came from Syn Innovation Laboratories S.A. (Athens, Greece) and [^177^Lu]LuCl_3_ (3.7 GBq/mL in 0.04 M HCl, RLu-3, A_s_ > 370 GBq/mg Lu) was provided by Mediray Ltd. (Athens, Greece).

#### 2.1.2. Radiolabeling–Radiochemistry

Lyophilized DT14D was dissolved in H_2_O (HPLC-grade) to achieve a concentration of 2 mg/mL. Equal 50 μL aliquots were placed in protein LoBind Eppendorf tubes (Merck SA, Athens, Greece) and stored at −20 °C. Labeling with Ga-67, In-111, and Lu-177 was performed, as detailed for each radionuclide in the Supplementary Materials. Likewise, the preparation of the respective metal-tagged DT14D analogs and pursuant HPLC analysis are described in the Supplementary Materials. Radioiodination of NT was conducted according to the chloramine-T method and the [^125^I]I-Tyr^3^-NT product was collected at high purity using HPLC, as previously described [56]. It was then diluted in 0.1% bovine serum albumin–phosphate-buffered saline (BSA-PBS) at a molar activity of 74 GBq/μmol, divided in equal aliquots in protein LoBind Eppendorf tubes, and stored at −20 °C.

Analysis of radiolabeled products was conducted using a Waters Chromatograph with a 600E multi-solvent delivery system coupled to a Waters 2998 photodiode array detector (Waters, Vienna, Austria) and a Gabi gamma-detector (Raytest, RSM Analytische Instrumente GmbH, Straubenhardt, Germany). Data processing and chromatographic runs were managed by Empower Software 2 (Waters, Milford, MA, USA). Radiolabeling reaction aliquots were loaded on a Symmetry Shield RP18 cartridge column (5 μm, 3.9 mm × 150 mm, Waters, Eschborn, Germany) and eluted with the following linear gradient: 100%A/0% B to 40%A/60% B in 20 min, whereby A = 0.1% TFA in H_2_O (*v*/*v*) and B = MeCN (system 1; representative chromatograms in Figure S1, Supplementary Materials).

Handling of solutions containing beta-/gamma-emitting radionuclides was conducted by authorized personnel in compliance with European radiation safety guidelines. Licensed facilities were supervised by the Greek Atomic Energy Commission (GAEC, license #A/435/17092/2019 and #A/435/15767/2019).

### 2.2. In Vitro Studies

#### 2.2.1. Cell Culture

The pancreatic adenocarcinoma AsPC-1 cell line was obtained from LGC Standards GmbH (Wesel, Germany) [57], whereas the colorectal adenocarcinoma WiDr cell line was purchased from LGC Promochem (Teddington, UK) [58]. AsPC-1 cells were grown in GLUTAMAX-I Roswell Park Memorial Institute-1640 (RPMI) and WiDr cells were grown in McCoy’s GLUTAMAX-I. Both culture media were supplemented with 10% (*v*/*v*) fetal bovine serum (FBS), 100 U/mL penicillin, and 100 µg/mL streptomycin. The cells were kept in 75 cm^2^ flasks with a vent cap at 37 °C in 95% humidity and 5% CO_2_-containing atmosphere in a Heal Force SMART CELL HF-90 incubator (Shanghai, China). When 75–85% confluency was reached, the cells were split at a 1:3–1:5 ratio by applying Trypsin/EDTA (0.05%/0.02% *w*/*v*) solution. Culture media were provided by Selidis, A., Bros—Antisel S.A. (Athens, Greece), while all supplements, the Trypsin/EDTA solution, and plastic consumables were provided by Bioline Scientific Douros Bros—E. Demagos O.E. (Athens, Greece).

#### 2.2.2. Competitive Binding of DT14D and Metal-Tagged DT14D to NTS_1_R

The affinity of metal-free DT14D and metal-tagged [^nat^Ga]Ga/[^nat^In]In/[^nat^Lu]Lu-DT14D (Supplementary Materials) for the human NTS_1_R was evaluated via competitive binding assays. These assays were performed using the [^125^I]I-Tyr^3^-NT radioligand in membrane homogenates obtained from WiDr cells, which were stored at −80 °C in 100 µL aliquots of Tris/EDTA buffer (10 mM Tris, 0.1 mM EDTA, pH 7.4) [56]. On the day of the experiment, the membrane homogenates were thawed, combined, and diluted in ice-cold binding buffer (BB: 50 mM HEPES, 5.5 mM MgCl_2_, 0.1 mg/mL bacitracin, 1% *w*/*v* BSA, pH 7.4). Each of the above ligands (including NT as a control) was incubated in triplicate at increasing concentrations (ranging from 10^−13^ M to 10^−6^ M in BB, 30 µL) with [^125^I]I-Tyr^3^-NT (214 pM in 70 µL BB) and membrane homogenate (300 µL in BB) for 1 h at 22 °C in an Incubator-Orbital Shaker unit (MPM Instr. SrI, Bernareggio, Milan, Italy). Following incubation, the samples were filtered rapidly through glass fiber filters (Whatman GF/B, Brandel Inc., Gaithersburg, MD, USA), presoaked in BB for at least 1 h, using a Brandel Cell Harvester (Adi Hassel Ingenieur Büro, Munich, Germany). The filters were washed with ice-cold washing buffer (WB, 10 mM HEPES pH 7.4, 150 mM NaCl), collected individually, placed in separate RIA tubes, and measured for their radioactivity content in a gamma counter (automated multi-sample well-type instrument with a NaI(Tl) 3″ crystal, Canberra Packard Cobra^TM^ Quantum U5003/1, Auto-Gamma^®^ counting system; Canberra Packard Central Europe GmbH, Schwadorf, Austria). The 50% inhibitory concentration (IC_50_) was determined with nonlinear regression analysis using a one-site model in PRISM^TM^ GraphPad Software (10.2 version, San Diego, CA, USA). The results are presented as the mean IC_50_ values ± standard deviation (sd) from three independent experiments, each performed in triplicate.

#### 2.2.3. NTS_1_R-Specific Internalization Experiments

The internalization of [^67^Ga]Ga/[^111^In]In/[^177^Lu]Lu-DT14D was studied in NTS_1_R-positive AsPC-1 cells [49,57]. The cells were seeded in 6-well plates (1 × 10^6^ per well) and left in the incubator overnight. On the following day, they were washed twice with ice-cold internalization medium (IM, RPMI- GLUTAMAX-I supplemented with 1% FBS) and the plates were left on the bench. To each well the following solutions were successively added: i. warm IM (1200 µL at 37 °C), ii. IM (150 µL; 3 upper wells: total series) or NT (10^−5^ M in IM, 3 bottom wells: non-specific series), and iii. radioligand (250 fmol in 150 µL IM). The plates were incubated at 37 °C for 1 h (in the Incubator-Orbital Shaker unit) and then placed on ice. Supernatants were collected in separate RIA tubes and cells were washed with ice-cold phosphate buffer saline (pH 7.4) supplemented with 0.5% *w*/*v* BSA (PBS-BSA; 1 mL); washings were collected and combined with the respective supernatants. The cells were next treated with acid glycine buffer (AGB, 50 mM glycine, 0.1 M NaCl, pH 2.8; 2 × 5 min with 600 µL) at ambient temperature and supernatants corresponding to the membrane bound fraction (MB) were collected. Cells were washed once again with ice cold PBS-BSA (1 mL), and subsequently lysed with 1 M NaOH (2 × 600 µL); the lysates were combined and collected (internalized fraction). The fractions obtained from each well collected in three respective RIA tubes (supernatant, MB and internalized) were measured for their radioactivity content on the γ-counter. The radioactivity of each fraction was then assessed as a percentage of the initial activity added per well. By subtracting the non-specific fractions from the respective total fractions, the specific MB and internalization fractions could be calculated. The results are expressed as the mean ± sd from at least three assays performed in triplicate using PRISM^TM^ GraphPad—10.2 Software (San Diego, CA, USA).

### 2.3. Animal Studies

#### 2.3.1. Metabolic Stability in Mice

The stability of [^67^Ga]Ga/[^111^In]In/[^177^Lu]Lu-DT14D radioligands was studied in the blood of mice collected 5 min post-injection (pi) and analyzed through HPLC analysis. Healthy male Swiss albino mice (16 animals in total, 30 ± 5 g, NCSR “Demokritos” Animal House, Athens, Greece) were used for the experiment. Animals received each radioligand with (2.5–3 nmol of total conjugate in vehicle–saline/EtOH 9/1 *v*/*v*, corresponding to up to 11 MBq for In-111, up to 13 MBq for Ga-67, and up to 74 MBq for Lu-177) or without (controls) or 30 min following oral gavage of Entresto^®^ (a slurry of 12 mg/200 µL per mouse, prepared as described in 2.1.1.; Entresto^®^). The animals were sacrificed at 5 min pi and blood was withdrawn from the heart in a heparinized pre-cooled penicillin syringe and transferred into a protein LoBind Eppendorf tube (containing 40 µL, 50 mM Na_2_EDTA solution) on ice. The collected blood samples underwent a previously described procedure to remove blood cells and proteins [56]. Aliquots of the final solution were analyzed for radiometabolite formation with radio-HPLC. An XTerra RP18 (5 µm, 3.9 mm × 20 mm) column (Waters, Eschborn, Germany) was eluted at a flow rate of 1 mL/min with the following gradient (system 2): 100% A/0% B to 60% A/40% B in 40 min; A = 0.1% TFA in H_2_O and B = MeCN. The results were finally expressed as average values ± sd from n independent experiments (for controls: n = 3; for Entresto^®^: [^111^In]In-DT14D, n = 3, [^67^Ga]Ga-DT14D, n = 2, and [^177^Lu]Lu-DT14D, n = 2). Statistical analysis was performed with PRISM^TM^ GraphPad—10.2 Software (San Diego, CA, USA), applying 2-way ANOVA with Tukey’s post hoc analysis; differences with *p* < 0.05 were considered as statistically significant.

#### 2.3.2. Biodistribution of [^67^Ga]Ga/[^111^In]In/[^177^Lu]Lu-DT14D in AsPC-1 Xenograft-Bearing Mice

The distribution of [^67^Ga]Ga/[^111^In]In/[^177^Lu]Lu-DT14D was investigated in male severe combined immunodeficiency (SCID) mice (49 mice in total, 6 weeks of age on arrival date, 22 ± 4 g, NCSR “Demokritos” Animal House, Athens, Greece) bearing NTS_1_R-positive AsPC-1 xenografts in their flanks. For xenograft induction, suspensions of freshly harvested AsPC-1 cells (5 × 10^6^ cells in 150 µL sterile PBS) were subcutaneously injected into the mice’s flanks. The animals were kept under aseptic conditions and 4 weeks later developed well palpable masses at the inoculation sites (100 ± 50 mg) and biodistribution was conducted. On the day of the experiment, the animals received a bolus (100 μL, in vehicle: saline/EtOH 9/1 *v*/*v*) containing the radioligand via the tail vein. The following groups of four animals per time point, per treatment, were included per radioligand (the 4 h block groups comprised 3 animals instead) including the respective (radio)ligand amounts injected: i. 78–91 kBq [^67^Ga]Ga-DT14D corresponding to 7–17 pmol DT14D at 1 and 4 h pi plus 4 h block (coinjection with 100 µg NT); ii. 74–92 kBq [^111^In]In-DT14D corresponding to 20–25 pmol at 4 h (controls), 4 h pi (Entresto^®^; animals receiving a slurry of 12 mg/200 µL Entresto^®^ oral gavage 30 min in advance) and 4 h block (coinjection with 100 µg NT); 24 h pi (including both controls and Entresto^®^ mice groups); and iii. 730 kBq [^177^Lu]Lu-DT14D corresponding to 40 pmol DT14D at 4, 24, 48, and 72 h pi plus 4 h block (co-injection with 100 µg NT). The animals were euthanized at predetermined time intervals, dissected and blood samples, organs of interest, and tumors were collected, weighed, and counted in the gamma counter. Biodistribution data were calculated as percent of the injected activity per gram tissue (%IA/g) with the aid of suitable standards of the administered activity. Results as the mean %IA/g values ± sd were obtained and statistical analysis was performed with PRISM^TM^ GraphPad—10.2 Software (San Diego, CA/USA), applying 2-way ANOVA with Tukey’s post hoc analysis, or, in the case of two variables, Šídák’s multiple comparisons test was adopted; differences with *p* < 0.05 were considered as statistically significant.

The animal studies were performed according to European and national regulations by trained and authorized personnel. The facilities were licensed (EL 25 BIO exp021) and the applied protocols were approved by the Department of Agriculture and Veterinary Service of the Prefecture of Athens (stability studies #440448, 1 June 2021; biodistribution/imaging studies #440451, 1 June 2021).

## 3. Results

### 3.1. Preparation of [^67^Ga]Ga/[^111^In]In/[^177^Lu]Lu-DT14D Radioligands

Radiolabeling of DT14D with Ga-67 (as a Ga-68 surrogate), In-111, and Lu-177 was successful following incubation of the respective radiometal chloride salts with a stock solution of DT14D at 80 °C, as detailed in the Supplementary Materials. As verified through radio-HPLC analyses (Figure S1, Supplementary Materials), the radioligands were obtained at a radiochemical purity of over 97% at apparent molar activities of 2.7–5.2 MBq [^67^Ga]Ga/nmol DT14D, 3.7–7.4 MBq [^111^In]In/nmol DT14D and 24–43 MBq [^177^Lu]Lu/nmol DT14D at the end of synthesis, respectively. Accordingly, the three radioligands were conveniently used without purification during the subsequent biological studies, and their integrity was always tested and confirmed prior to and at the end of each separate experiment.

### 3.2. In Vitro Characterization

#### 3.2.1. NTS_1_R Affinity of DT14D and [^nat^Ga]Ga/[^nat^In]In/[^nat^Lu]Lu-DT14D

**Figure 2:**
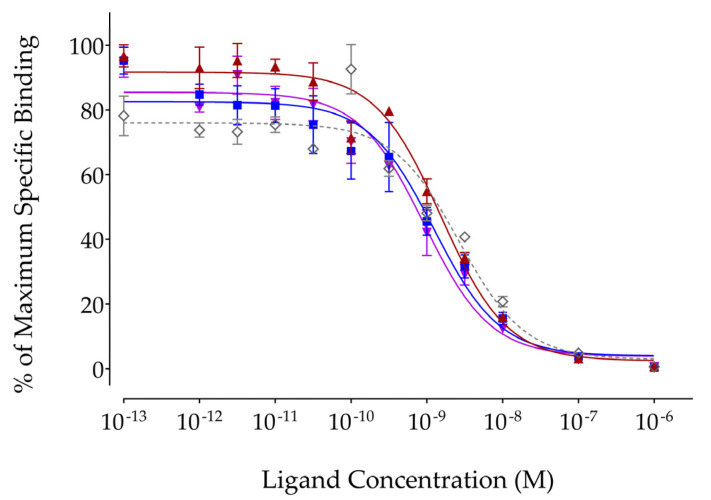
Displacement of [^125^I]I-Tyr^3^-NT from NTS_1_R binding sites in WiDr cell membranes with increasing concentrations of DT14D—grey dashed line (◇, IC_50_ = 2.56 ± 0.26 nM, n = 3), [^nat^Ga]Ga-DT14D—red line (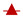, IC_50_ = 1.55 ± 0.14 nM, n = 3), [^nat^In]In-DT14D—blue line (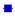, IC_50_ = 1.37 ± 0.54 nM, n = 3), and [^nat^Lu]Lu-DT14D—violet line (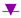, IC_50_ = 0.99 ± 0.18 nM, n = 3); the results represent the mean IC_50_ values ± sd, n = number of separate experiments in triplicate.

The affinity of DT14D and its Ga, In, and Lu tagged versions for the human NTS_1_R was determined using competitive binding assays against [^125^I]I-Tyr^3^-NT. The preparation of the three metal-tagged DT14D is detailed in the Supplementary Materials and analytical HPLC data on their formation (*t*_R_ shift of at least 1 min from the parent DT14D) are summarized in Table S2 (Supplementary Materials). Comparative curves of radioligand displacement from NTS_1_R-sites in WiDr membrane homogenates by increasing concentrations of DT14D and its Ga-, In-, and Lu-tagged versions are shown in Figure 2.

The following rank of increasing NTS_1_R affinity could be stablished: DT14D (IC_50_ = 2.56 ± 0.26 nM) < [^nat^Ga]Ga-DT14D (IC_50_ = 1.55 ± 0.14 nM) < [^nat^In]In-DT14D (IC_50_ = 1.37 ± 0.54 nM) < [^nat^Lu]Lu-DT14D (IC_50_ = 0.99 ± 0.18 nM). Incorporation of the metal seemed to have a positive effect on receptor affinity but was found to be statistically significant only for In (*p* < 0.05) and Lu (*p* < 0.01) but not for Ga (*p* > 0.05). This finding may be related to the reduction in negative charges of the pendant carboxylate arms of DOTA being engaged in metal binding. The NTS_1_R affinity differences among the metal-tagged DT14D species were not found to be statistically significant either (*p* > 0.05).

#### 3.2.2. Internalization of [^67^Ga]Ga/[^111^In]In/[^177^Lu]Lu-DT14D in AsPC-1 Cells

**Figure 3:**
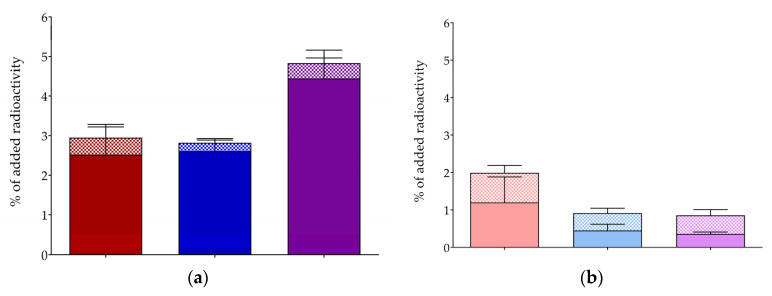
(**a**) NTS_1_R-specific uptake (specific internalized (solid), + specific membrane-bound (checkered) = total specific cell-associated) of [^67^Ga]Ga-DT14D (red bars), [^111^In]In-DT14D (blue bars) and [^177^Lu]Lu-DT14D (violet bars) and (**b**) non-specific (ns) uptake (ns internalized (solid) + ns membrane-bound (checkered) = total ns cell-associated) of [^67^Ga]Ga-DT14D (light red bars), [^111^In]In-DT14D (light blue bars), and [^177^Lu]Lu-DT14D (light violet bars) during 1 h incubation at 37 °C with confluent monolayers of AsPC-1 cells. The results are expressed as average values ± sd from 3 independent experiments, each performed in triplicate.

The uptake and internalization of [^67^Ga]Ga/[^111^In]In/[^177^Lu]Lu-DT14D in AsPC-1 confluent monolayers was studied during 1 h incubation at 37 °C, either alone (total) or in the presence of 1 mM NT for NTS_1_R blockade (non-specific, ns), and the specific values were determined by subtracting the ns from the totals for each well triplicate. Cumulative specific and ns results are presented in Figure 3 (a and b, respectively), revealing clear NTS_1_R-mediated cell uptake for all three radioligands. In all cases, we observed that the bulk of cell-associated activity was internalized in the cells with only a small fraction thereof still bound on the cell membrane, consistent with a receptor agonist profile.

### 3.3. Studies in Animals

#### 3.3.1. Metabolic Stability in Mice

**Figure 4:**
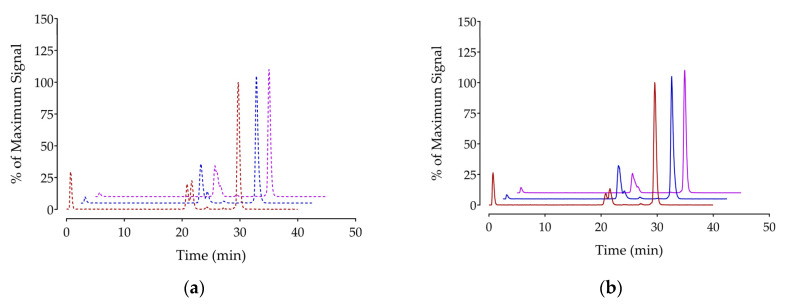
Representative radiochromatograms of HPLC analysis (system 2) of mouse blood samples collected 5 min pi of (**a**) radiolabeled DT14D (red for [^67^Ga]Ga-DT14D, blue for [^111^In]In-DT14D, and violet for [^177^Lu]Lu-DT14D) administered alone (dashed lines; controls) or (**b**) 30 min after oral gavage of Entresto^®^ (solid lines; Entresto^®^); intact radioligand percentages are included in Table 1.

The in vivo stability of [^67^Ga]Ga/[^111^In]In/[^177^Lu]Lu-DT14D was studied through HPLC analysis of blood collected 5 min following radioligand injection in healthy mice for radiometabolite formation (controls). Potential involvement of NEP in their catabolism could be established using separate groups of animals treated with Entresto^®^ 30 min in advance of radioligand injection (Entresto^®^ group) [56]. Representative radiochromatograms of this study are presented in Figure 4 (a and b, respectively), whereas cumulative stability outcomes are summarized in Table 1, whereby results previously reported for the [^99m^Tc]Tc-DT11 parent are also included to facilitate direct comparisons [56].

It is interesting to observe that the three new radioligands displayed significantly higher in vivo stability compared with [^99m^Tc]Tc-DT11 ([^111^In]In-DT14D or [^177^Lu]Lu-DT14D, *p* < 0.001; [^67^Ga]Ga-DT14D, *p* < 0.0001), implying that the metal–chelate affects in vivo stability. The involvement of NEP in their in vivo degradation, although visible, was less pronounced than for [^99m^Tc]Tc-DT11 (*p* < 0.0001) [56]. Thus, the improvement observed in the Entresto^®^-treated group vs. controls in the case of the most stable [^67^Ga]Ga-DT14D was found to be non-statistically significant (*p* > 0.05), unlike for [^111^In]In-DT14D and [^177^Lu]Lu-DT14D, which displayed statistically significant stability improvements (*p* < 0.01) [56].

#### 3.3.2. Biodistribution of [^67^Ga]Ga/[^111^In]In/[^177^Lu]Lu-DT14D in Mice Bearing AsPC-1 Tumors

**Figure 5:**
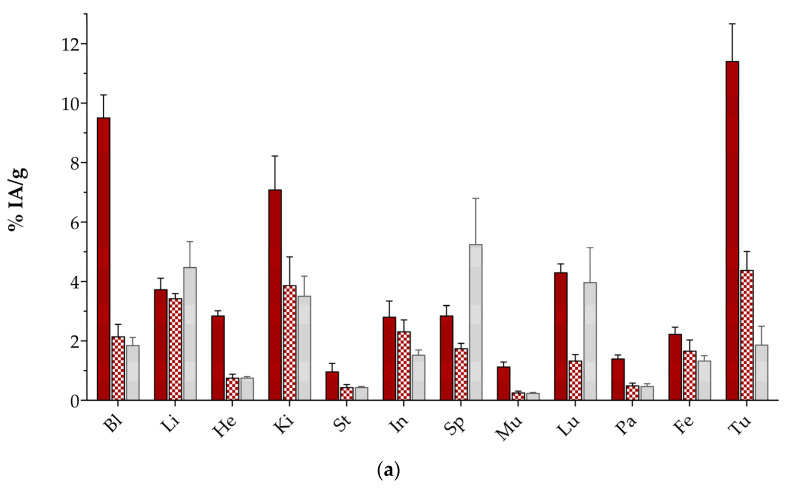

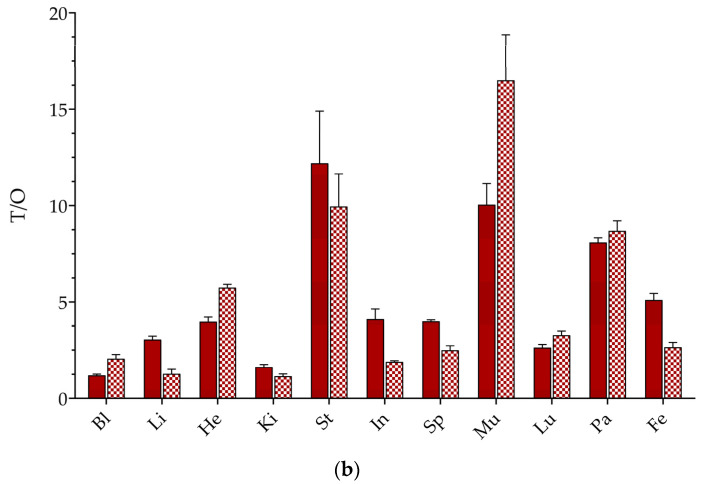
Biodistribution of [^67^Ga]Ga-DT14D in SCID mice with AsPC-1 tumors at 1 h (red bars) and 4 h pi (red checkered bars—controls; and blocked (treated with excess NT)—light gray solid bars); (**a**) data represent average %IA/g values ± sd, n = 4 and (**b**) tumor-to-organ (T/O) ratios; Bl = blood, Li = liver, He = heart, Ki = kidneys, St = stomach, In = intestines, Sp = spleen, Mu = muscle, Lu = lung, Pa = pancreas, Fe = femur, and Tu = AsPC-1 xenografts.

**Figure 6:**
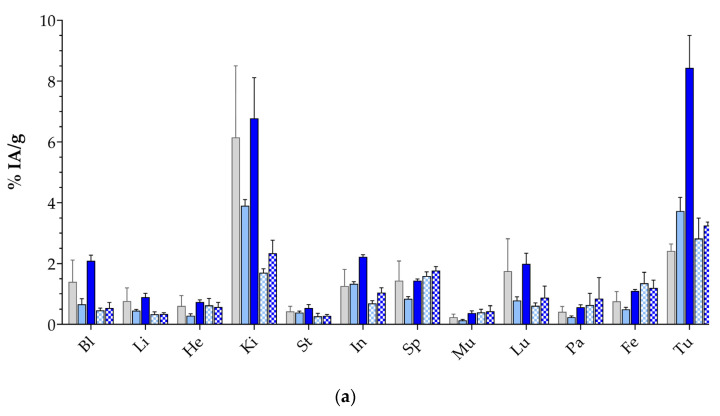

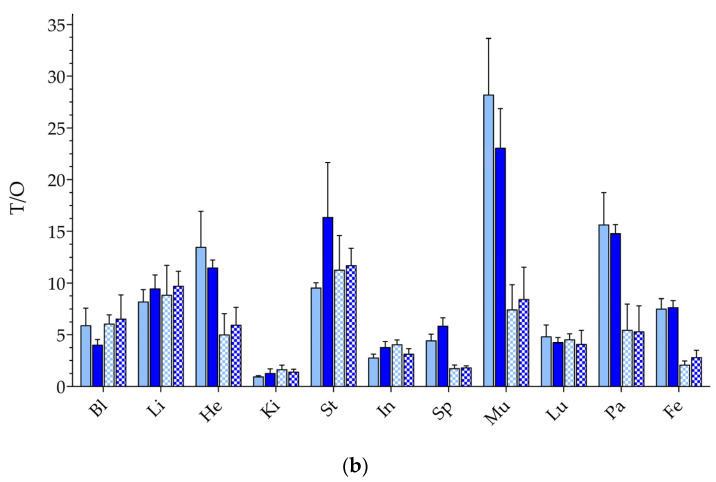
Biodistribution of [^111^In]In-DT14D in SCID mice bearing AsPC-1 xenografts from left to right: 4 h block (1st light gray solid bars—treated with excess NT), 4 h controls (2nd light blue solid bars), 4 h Entresto^®^-treated mice (3rd darker blue solid bars), 24 h controls (light blue checkered bars), and 24 h pi Entresto^®^-treated mice (darker blue checkered bars); (**a**) data represent average %IA/g values ± sd, n = 4 and (**b**) tumor-to-organ (T/O) ratios (4 h block—1st light gray solid bars not included); Bl = blood, Li = liver, He = heart, Ki = kidneys, St = stomach, In = intestines, Sp = spleen, Mu = muscle, Lu = lung, Pa = pancreas, Fe = femur, and Tu = AsPC-1 xenografts.

**Figure 7:**
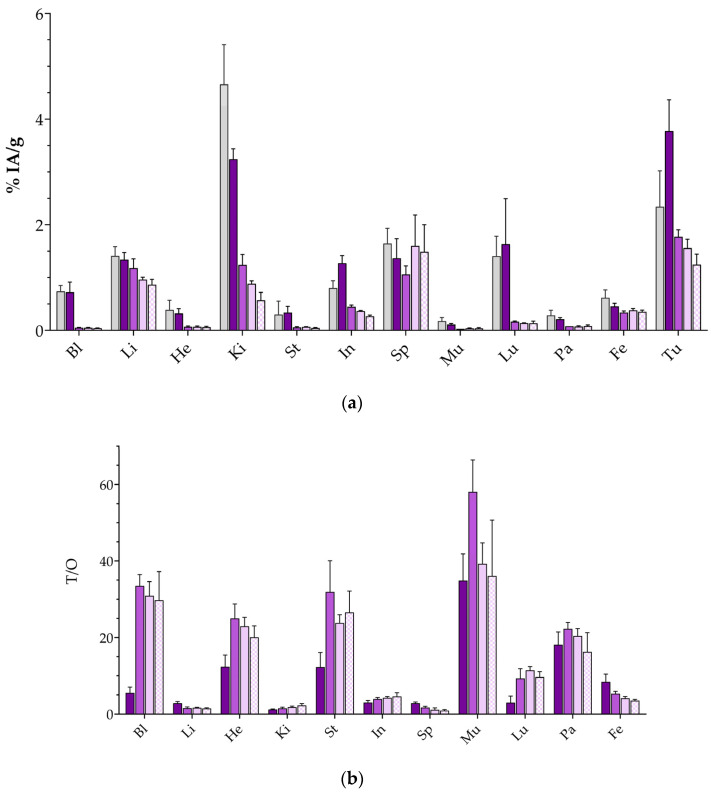
Biodistribution of [^177^Lu]Lu-DT14D in SCID mice bearing AsPC-1 xenografts from left to right: 4 h block (1st light gray solid bars—treated with excess NT), 4 h (2nd dark violet solid bars), 24 h (3rd lighter violet solid bars), 48 h (4th very light violet solid bars), and 72 h pi (5th very light violet chequered bars). The 4–72 h pi values refer to controls (non-treated with Entresto^®^); (**a**) data represent average %IA/g values ± sd, n = 4 and (**b**) tumor-to-organ (T/O) ratios (4 h block—1st light gray solid bars not included); Bl = blood, Li = liver, He = heart, Ki = kidneys, St = stomach, In = intestines, Sp = spleen, Mu = muscle, Lu = lung, Pa = pancreas, Fe = femur, and Tu = AsPC-1 xenografts.

The biodistribution results of [^67^Ga]Ga/[^111^In]In/[^177^Lu]Lu-DT14D radioligands in mice bearing human AsPC-1 xenografts expressing the NTS_1_R are summarized in Figure 5, Figure 6 and Figure 7, respectively, with numerical data presented in Tables S3–S5 (Supplementary Materials). Data are provided as average %IA/g values ± sd at several time points and for several treatment groups, as will be individually discussed for each analog below.

Firstly, the biodistribution of [^67^Ga]Ga-DT14D was studied at 1 h and 4 h pi, taking into account the short half-life of Ga-68 (t_1/2_ of 68 min) for which Ga-67 served as a surrogate in the present work. As shown in Figure 1a (Table S3a—Supplementary Materials), impressively high uptake of the radiotracer was achieved at 1 h pi (11.42 ± 1.25 %IA/g), which rapidly declined, however, at 4 h pi (4.39 ± 0.62 %IA/g; *p* < 0.0001). The latter tumor uptake was shown to be NTS_1_R-specific by the significant reduction observed in the mice receiving an excess amount of NT (1.88 ± 0.62 %IA/g; *p* < 0.0001). Another important finding is that the initial high tumor uptake of [^67^Ga]Ga-DT14D was accompanied by unfavorable background activity in most tissues and organs of the body, potentially related to the high values found in the blood at 1 h pi (9.52 ± 0.76 %IA/g). These values subsequently dropped at 4 h pi, resulting in an increase in most of the T/O ratios (Figure 5b, Table S3b—Supplementary Materials). Although the T/O ratio increase was not statistically significant in most cases (e.g., the Tu-to-Bl ratio increased from 1.20 ± 0.06 to 2.06 ± 0.21; *p* > 0.05), the respective increase in the Tu-to-Mu ratio turned out to be prominent at 4 h pi (10.05 ± 1.09 to 16.50 ± 2.36; *p* < 0.0001).

The biodistribution of [^111^In]In-DT14D in the same animal model was studied next. According to the in vivo stability data, treatment of mice with Entresto^®^ significantly improved the in vivo stability of [^111^In]In-DT14D, unlike [^67^Ga]Ga-DT14D not showing significant stability improvements during NEP inhibition induced by Entresto^®^. Furthermore, the longer half-life of In-111 (t_1/2_ 67.2 h) compared with the clinically relevant [^68^Ga]Ga-DT14D (represented herein by [^67^Ga]Ga-DT14D) renders longer time intervals much more interesting, whereby lower background activity is anticipated. Therefore, the biodistribution of [^111^In]In-DT14D was studied at 4 and 24 h pi, without or during treatment with Entresto^®^, and the results are presented in Figure 6 and in Table S4 (Supplementary Materials).

First of all and in line with the in situ stabilization effect, we observed a significant increase of tumor uptake at 4 h pi in the Entresto^®^-treated animals (3.73 ± 0.45 %IA/g—controls, to 8.44 ± 1.06 %IA/g—Entresto^®^; *p* < 0.0001), which could be blocked with excess NT (to 2.42 ± 0.23 %IA/g—block; *p* < 0.01 vs. controls and *p* < 0.0001 vs. Entresto^®^), as consistent with an NTS_1_R-mediated process. The Entresto^®^ effect turned out to be non-significant at 24 h pi (from 2.82 ± 0.68 %IA/g—controls, to 3.25 ± 0.11 %IA/g—Entresto^®^; *p* > 0.05). Compared with the 4 h Entresto^®^ group, tumor values significantly declined independent of treatment at 24 h pi (*p* < 0.0001 both in the Entresto^®^ and control groups). In contrast, compared with the 4 h controls, tumor values dropped only slightly in the 24 h control group (*p* < 0.05), while remaining unchanged in the 24 h Entresto^®^ group (*p* > 0.05). We also observed consistent increases in healthy organ activity at 4 h pi between the controls and Entresto^®^-treated mice, as, for example, in the kidneys (3.90 ± 0.20 %IA/—controls to 6.78 ± 1.33 %IA/g—Entresto^®^; *p* < 0.001). Therefore, for most organs, the T/O ratios neither changed nor improved (Figure 6b and Table S4b Supplementary Materials).

Eventually, the biodistribution of [^177^Lu]Lu-DT14D in mice bearing human AsPC-1 tumors was studied next at 4, 24, 48, and 72 h pi, in view of the longer half-life of Lu-177 (t_1/2_ 6.7 d). On the other hand, taking into account that no clear benefits were revealed in the T/O ratios during NEP-inhibition conditions in the [^111^In]In-DT14D study, additional Entresto^®^-treated animal groups were not included in the [^177^Lu]Lu-DT14D experiment. Results of the latter are summarized in Figure 7 and in Table S5 (Supplementary Materials).

## 4. Discussion

The search for suitable NTS_1_R-targeting radioligands for diagnostic and internal radiotherapy of human tumors has been intense, prompted by the overexpression of NTS_1_R documented in a multitude of human cancers [7,8,9,10,12,13,14,15,16,18,19,20,22,23,30,58]. A good number of radiolabeled NT(8-13) analogs have been developed over the years [24,25,27,28,29,30,32,33,34]. Yet, candidates of choice have thus far led to rather disappointing outcomes during clinical translation studies in patients with NTS_1_R-expressing tumors, presumably as a result of fast degradation in the biological milieu [36,37,38]. Amongst the proteases reported for their proteolytic action on NT [39,41,42,45], NEP and ACE have been shown to play a key-role in the rapid degradation of NT(8-13)-based radioligands injected into mice [44,49,55]. In fact, we have also confirmed the combined and fast degrading power of these two key proteases while studying the metabolic stability of our prototype [^99m^Tc]Tc-DT1 (DT1, N_4_-Gly-Arg-Arg-Pro-Tyr-Ile-Leu-OH), and a series of mimics thereof in mice blood collected 5 min pi without or during NEP/ACE inhibition. We were able to show strong stabilization of these analogs through the administration of NEP/ACE inhibitor regimens. For example, while only 1.8 ± 0.8% of [^99m^Tc]Tc-DT1 remained intact in mice circulation at 5 min pi, this percentage impressively increased as high as 63.8 ± 7.5% during combined NEP/ACE inhibition. Most importantly, this inhibitor-induced stabilization, shown at 5 pi, led to significant enhancement of uptake in AsPC-1 xenografts in mice at 4 h pi (from 1.25 ± 0.14 %IA/g in controls to 7.05 ± 0.8 %IA/g in the NEP/ACE-treated mice) [49]. These in vivo results, corroborated by a multitude of studies in other peptide radioligands, highlight the need for stability assessments conducted in vivo, taking into account that NEP is a very-fast-acting omnipresent ectoenzyme with a wide substrate repertoire, scarcely present in serum or plasma [43,59].

Aiming to achieve metabolic stability improvements, we have recently introduced [^99m^Tc]Tc-DT11, a branched [^99m^Tc]Tc-DT1 mimic carrying the pendant MPBA-PEG4 chain at the *ε*-amine of Lys^7^ of the N_4_-Lys-Arg-Arg-Pro-Tyr-Ile-Leu-OH backbone (Figure 1a). This modification led to significantly better in vivo stability vs. the [^99m^Tc]Tc-DT1 parent, especially in terms of resistance to ACE, whilst other important biological traits, such as receptor affinity, cell internalization, good tumor targeting, and pharmacokinetics were not negatively affected [55,56]. Single in-situ inhibition of NEP through the administration of specific NEP inhibitors to mice further improved the in vivo stability and the biodistribution profile of [^99m^Tc]Tc-DT11, presenting promising prospects for clinical translation by simplifying clinical protocols and accordingly clinical study approvals [56]. Open chain tetraamines though are mostly suited for labeling with Tc-99m in view of the distinctive coordination chemistry of this group 7 element [60]. Consequently, the clinical potential of [^99m^Tc]Tc-DT11 is actually restricted in the diagnostic imaging of NTS_1_R-positive tumors with SPECT. Aiming to broaden this potential toward radiotheranostics, we proceeded in “reshaping” [^99m^Tc]Tc-DT11 to DT14D. The latter is functionalized instead with the universal chelator DOTA attached via three βAla-residues to the peptide (Figure 1b), which allows for labeling with a wide palette of trivalent radiometals for SPECT, PET, and internal radiotherapy [31]. In this work, we compared a representative series of [^67^Ga]Ga/[^111^In]In/[^177^Lu]Lu-DT14D radioligands with the [^99m^Tc]Tc-DT11 parent and were thus able to draw significant conclusions for improving the design of new branched NT(8-13) analogs for effective NTS_1_R-directed cancer radiotheranostics.

Firstly, this study revealed a substantial reduction in receptor affinity in DT14D and its Ga/In/Lu-tagged versions (Figure 2) compared with DT11 (IC_50_ = 0.08 ± 0.05 nM), determined under the same experimental settings, namely during competitive binding assays against the [^125^I]I-Tyr^3^-NT radioligand in NTS_1_R-rich WiDr cell membrane homogenates [56]. Furthermore, metal-tagged DT14D displayed significantly higher NTS_1_R affinity compared with DT14D (Figure 2). This finding seems to be linked with the significant reduction in negative charges when the deprotonated pendant carboxylate arms of DOTA are engaged in coordination with the metal. All of the above observations corroborate the assumption that positive charge(s) at the N-terminus of the molecule has/have a favorable effect on receptor affinity, an observation also made in recent reports [33,61]. Likewise, a considerable drop in the NTS_1_R-specific internalization of [^67^Ga]Ga/[^111^In]In/[^177^Lu]Lu-DT14D in AsPC-1 cells was observed (Figure 3) in comparison with [^99m^Tc]Tc-DT11 (13.4 ± 0.8% at 1 h incubation at 37 °C) [56], following the receptor affinity trend.

A second important finding of the present study was the switch of the monocationic *trans*-[^99m^Tc][Tc(O)_2_(N_4_)]^+^ metal-chelate of [^99m^Tc]Tc-DT11 by any of the three DOTA-radiometal-chelates of [^67^Ga]Ga/[^111^In]In/[^177^Lu]Lu-DT14D had the opposite effect on in vivo stability (Table 1). Thus, all three DT14D radioligands turned out to be significantly more stable than [^99m^Tc]Tc-DT11 in peripheral mice blood, according to HPLC analysis of collected blood samples (Table 1) [56] and impressively higher than the [^99m^Tc]Tc-DT1 prototype (1.8 ± 0.8% intact at 5 min pi) [44]. Furthermore, treatment of mice with Entresto^®^ exerted a considerably smaller stabilization effect on [^67^Ga]Ga/[^111^In]In/[^177^Lu]Lu-DT14D compared with the stability improvement previously reported for [^99m^Tc]Tc-DT11 [56]. These findings demonstrate a marked resistance of the branched [^67^Ga]Ga/[^111^In]In/[^177^Lu]Lu-DT14D analogs not only to ACE but also to NEP.

The impact of the above opposing results on tumor targeting and biodistribution of [^67^Ga]Ga/[^111^In]In/[^177^Lu]Lu-DT14D in mice bearing AsPC-1 xenografts was compared next with [^99m^Tc]Tc-DT11, previously conducted in the same animal model [56]. At first, it is interesting to observe that the tumor uptake values at 4 h pi are very similar across the analogs (for Ga-67: 4.39 ± 0.62 %IA/g; for In-111: 3.73 ± 0.45 %IA/g; for Lu-177: 3.77 ± 0.59 %IA/g) and compared with [^99m^Tc]Tc-DT11 (4.48 ± 0.37 %IA/g) [56], revealing that the reduction in NTS_1_R affinity and in vitro cell internalization of [^67^Ga]Ga/[^111^In]In/[^177^Lu]Lu-DT14D have been counterbalanced by their higher in vivo stability vs. their Tc-99m parent. This notion was further corroborated by the tumor uptake values at 24 h pi, which were also found at quite similar levels between [^111^In]In/[^177^Lu]Lu-DT14D (for In-111: 2.11 ± 0.34 %IA/g; for Lu-177: 1.77 ± 0.13 %IA/g) and compared with [^99m^Tc]Tc-DT11 (1.87 ± 0.07 %IA/g) [56]. It should be noted that uptake in most physiological organs and tissues of the mice was also very similar between these two groups of radioligands.

Another interesting outcome of the present study concerns the effect of Entresto^®^ on biodistribution patterns. These were investigated in the case of [^111^In]In-DT14D, compared with [^99m^Tc]Tc-DT11, and related to Entresto^®^-induced stabilization effects. Thus, treatment of mice with Entresto^®^ led to a marked increase in tumor values for [^111^In]In-DT14D at 4 h pi (to 8.44 ± 1.06 %IA/g) surpassing the respective uptake increase in the [^99m^Tc]Tc-DT11 parent (to 6.14 ± 0.08 %IA/g) in the same animal model. This improvement should not be attributed exclusively to the rather minor stabilization of [^111^In]In-DT14D in mice blood at 5 min pi (≈10% stability improvement; Table 1), however, considering that the respective Entresto^®^-induced stabilization of [^99m^Tc]Tc-DT11 was clearly more pronounced (>35% stability enhancement; Table 1) [56]. The unexpectedly higher increase in the tumor uptake of [^111^In]In-DT14D seems to be rather related to the higher radioactivity values in the blood of the Entresto^®^-treated mice. Higher blood levels led to higher uptake in several physiological tissues too, eventually preventing favorable increases in the T/O ratios in the Entresto^®^ group (Figure 6). Based on these results, the biodistribution study of [^177^Lu]Lu-DT14D did not include additional mice groups treated with Entresto^®^ (Figure 7). Of particular interest in the biodistribution of [^177^Lu]Lu-DT14D turned out to be the persistent tumor retention of the radioligand in the interval between 24 to 72 h pi, an attractive feature as far as radionuclide therapy with the longer-lived beta emitter is considered. The therapeutic efficacy of [^177^Lu]Lu-DT14D warrants further investigation in dedicated therapy studies, whereby regimens with different amounts of injected peptide mass are investigated and careful dosimetric calculations follow to select the most favorable protocols for maximum therapeutic outcomes.

In conclusion, replacement of the open chain tetraamine of the branched [^99m^Tc]Tc-DT11 radioligand with the universal chelator DOTA coupled via the (βAla)_3_-spacer has allowed labeling with medically relevant trivalent radiometals. Representative radioligands of DT14D with Ga-67 (a surrogate of Ga-68: PET), In-111 (SPECT), and Lu-177 (radiotherapy) investigated in NTS_1_R-positive models were compared with the [^99m^Tc]Tc-DT11 parent revealing the importance of positive charge at the N-peptide terminus for radioligand affinity. The increased metabolic stability of the three [^67^Ga]Ga/[^111^In]In/[^177^Lu]Lu-DT14D analogs resulted not only due to the lateral chain attached to the ε-amine of Lys^7^ of these radioligands (also present in [^99m^Tc]Tc-DT11) but due to the replacement of the monocationic *trans*-[^99m^Tc][Tc(O)_2_(N_4_)]^+^-chelate of [^99m^Tc]Tc-DT11 tethered at the A-amine of Lys^7^ by each of the [^67^Ga]Ga/[^111^In]In/[^177^Lu]Lu-DOTA-(βAla)_3_ residues. The tumor uptake of [^67^Ga]Ga/[^111^In]In/[^177^Lu]Lu-DT14D turned out to be in the same range as the [^99m^Tc]Tc-DT11 reference, highlighting the additional and equally crucial role of in vivo stability in biodistribution patterns. It is rational to assume that the introduction of positive charges in DT14D through the replacement of one or more βAla in the linker by basic residues (e.g., Pip, 4-amino-1-carboxymethyl-piperidine) [29,62] may represent an elegant way to combine the above important features of increased receptor affinity and in vivo stability in one molecule. Another smart approach inherently evading the interference of rapidly degrading proteases on radioligand efficacy is the development of non-peptidic analogs to target NTS_1_R-positive lesions. In fact, such radioligands displaying receptor-antagonist properties have been recently proposed and their value in NTS_1_R-positive cancer theranostics is currently being explored [63,64,65,66].

## Figures and Tables

**Table 1:** Stabilities of [^67^Ga]Ga-DT14D, [^111^In]In-DT14D, and [^177^Lu]Lu-DT14D in mice blood samples collected 5 min pi. Non-treated animal groups (controls) and groups of mice treated with Entresto^®^ (Entresto^®^) are separately shown in the Table. The respective data for [^99m^Tc]Tc-DT11 used as reference are also included (adapted from [56]).

	[^67^Ga]Ga-DT14D	[^111^In]In-DT14D	[^177^Lu]Lu-DT14D	[^99m^Tc]Tc-DT11
Control	73.1 ± 3.3 (3)	69.5 ± 0.7 (3)	68.4 ± 2.1 (3)	56.56 ± 5.19 (3)
Entresto^®^	79.8 ± 0.3 (2)	79.5 ± 3.1 (3)	79.5 ± 0.7 (2)	76.98 ± 3.31 (3)

Data represent the mean percentage of intact radioligand ± sd with the number of experiments shown in parentheses.

## Data Availability

Data are contained within the article and in the Supplementary Material.

## Supplementary Materials

The following supporting information can be downloaded at: https://www.mdpi.com/article/10.3390/pharmaceutics17030310/s1, Figure S1: HPLC analysis of [^67^Ga]Ga-DT14D, [^111^In]In-DT14D and [^177^Lu]Lu-DT14D; Table S1. Analytical data for DT14D; Table S2: Analytical HPLC data for (radio)metal-tagged TD14D; Table S3: Biodistribution of [^67^Ga]Ga-DT14D; Table S4: Biodistribution of [^111^In]In-DT14D; Table S5: Biodistribution of [^177^Lu]Lu-DT14D.
