# Supplementary material for: Reshaping [99mTc]Tc-DT11 to DT14D Tagged with Trivalent Radiometals for NTS1R-Positive Cancer Theranostics
Source: Pharmaceutics. 2025 Feb 28;17(3):310. doi: 10.3390/pharmaceutics17030310 (PMC11944670; doi:10.3390/pharmaceutics17030310)
Supplement: Supplementary file 1 [file pharmaceutics-17-00310-s001.zip › pharmaceutics-3470989-supplementary.pdf]

pharmaceutics

MDPI

Article

Supplementary Materials

Reshaping [^{99m}Tc]Tc-DT11 to DT14D Tagged with Trivalent Radiometals for NTS$_1$R-Targeted Cancer Theranostics

Panagiotis Kanellopoulos, Berthold A. Nock, Eric P. Krenning and Theodosia Maina *

Analytical data for DT114D

Analytical data for DT14D ([DOTA-βAla-βAla-βAla-Lys(MPBA-PEG4)^7-Arg-Arg-Pro-Tyr-Ile-Leu-OH; DOTA, 1,4,7,10-tetraazacyclododecane-1,4,7,10-tetraacetic acid; MPBA, (4-(4-methylphenyl)butyric acid); PEG4, 14-amino-3,6,9,12-tetraoxatetradecan-1-oic acid) from PiChem Forschungs- und Entwicklungs GmbH (Raaba-Grambach, Austria), comprising purity via HPLC analysis and MALDI-TOF data is summarized in Table S1.

Table S1. Analytical data for DT14D.

	HPLC				MW [c] calcd	MW found [d], m/z
	t_R (min)		% Purity			
DT14D	13.0 [a]	18.8 [b]	>95 [a]	>99 [b]	1938.3	1939.2

[a] A Nucleosil C18 (5 µm, 4 mm × 150 mm) column (MACHEREY-NAGEL GmbH & Co. KG; Dueren, Germany) was eluted at 1 mL/min flow rate with the following gradient: 90%A/10%B to 10%A/90%B in 30 min, UV trace at 215 nm; A: 0.1% TFA, B: 0.1%TFA in MeCN. [b] A Symmetry Shield RP-18 (5 µm, 3.9 mm × 20 mm) cartridge column (Waters, Vienna, Austria) was eluted at a 1 mL/min flow rate with the following linear gradient (system 1-A): from 100%A/0%B to 80% A/20% B in 5 min and to 72.5%A/27.5%B in 5 min in 30 min; A = 0.01% TFA and B = MeCN – UV trace at 220 nm; [c] average mass; [d] verification on MALDI TOF mass spectrometry.

Labeling of DT14D with Ga-67, In-111 and Lu-177

Labeling with Ga-67. A series of items was successively added to an 1.5 mL Eppendorf LoBind tube: i. EtOH (40 µL), ii. [^{67}Ga]GaCl$_3$ (27 µL; 4 – 5.5 GBq/mL in dilute HCl), iii. a freshly prepared solution of sodium para-aminobenzoic acid (Na-PABA, 10 µL, 0.5 M) in bi-distilled water, iv. DT14D stock solution (25 µL, 50 µg, ≈25 nmol) and sodium acetate buffer (10 µL, pH 4, 1 M). After a 30 min incubation at 80 °C, a 2 µL aliquot was withdrawn and quenched with 28 µL of an acetate buffered solution of disodium ethylenediaminetetraacetic acid (Na$_2$-EDTA, 1 mM, pH 4.0). [^{67}Ga]Ga-DT14D was obtained in radiochemical purities exceeding 97% at apparent molecular activities of 2.7 – 5.2 MBq [^{67}Ga]Ga/nmol DT14D, as shown in the representative radiochromatogram in Figure S1-a.

Labeling with In-111. The above protocol was slightly modified with the following series of items added to the Eppendorf LoBind tube: i. EtOH (100 µL), ii. [^{111}In]InCl$_3$ (150 µL, 370 – 740 MBq/mL in 0.05 M HCl), iii. a freshly prepared Na-PABA solution (25 µL, 0.5 M), iv. DT14D stock solution (25 µL, 50 µg, ≈25 nmol), and v. sodium acetate buffer (15 µL, pH 4.0, 1 M). The labeling reaction mixture was incubated at 80 °C for 30 min, DTPA was added as a scavenger of unbound In-111 and radioanalytical HPLC was performed, showing the formation of [^{111}In]In-DT14D in radiochemical purities exceeding 97% at apparent molecular activities of 3.7 – 7.4 MBq [^{111}In]In/nmol DT14D; a representative radiochromatogram is included in Figure S1-b.

Labeling with Lu-177. For Lu-177 labeling of DT14D the above protocol was adopted, as follows: i. EtOH (120 µL), ii. [^{177}Lu]$LuCl_3$ (150 µL, 3.7 GBq/mL in 0.04 M HCl, A_s > 370 GBq/mg Lu), iii. sodium acetate buffer (30 µL pH 4.6, 1.0 M), iv. a freshly prepared Na- PABA solution (30 µL, 0.5 M), and v. DT14D stock solution (35 µL, 70 µg, ≈35 nmol). The mixture was heated at 80 °C for 20 min. DTPA was added as a scavenger of unbound Lu- 177 and radioanalytical HPLC was performed and confirmed the formation of [^{177}Lu]Lu- DT14D in high radiochemical purities (>97 %) at apparent molar activities of 24-43 MBq [^{177}Lu]Lu/nmol DT14D at the end of synthesis; a representative radiochromatogram is in- cluded in Figure S1-c.

In view of the high purity formation of forming radioligands, no further purification was required for subsequent biological testing. For all further in vitro and in vivo studies, the labeling solution was diluted to the desired activity concentration using a 5 mM solu- tion of Na-PABA dissolved in phosphate buffered saline. The integrity of radiotracers was tested before and after the conclusion of all biological experiments and was found pre- served in all cases.

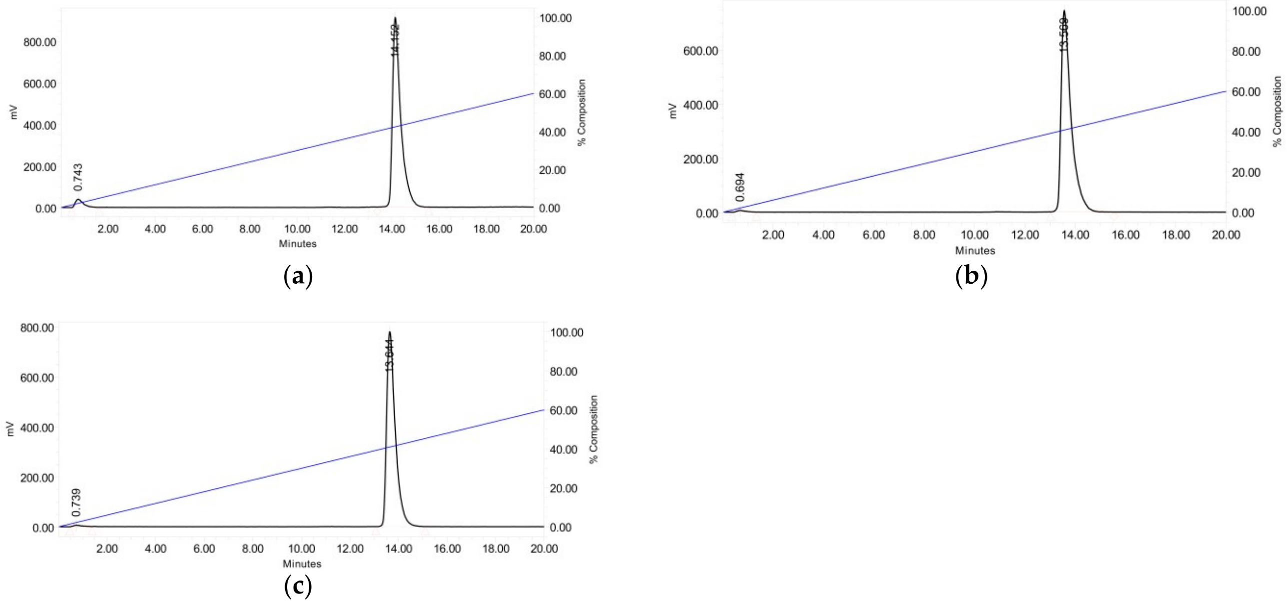

Figure S1. Representative radiochromatograms of HPLC analysis of (**a**) [^{67}Ga]Ga-DT14D (t_R, 14.15 min; 97.2%) (**b**) [^{111}In]In-DT14D (t_R, 13.57 min; 99.2%) and (**c**) [^{177}Lu]Lu-DT14D (t_R, 13.64 min; 99.3%) radiolabeled product, verifying the >97% formation of a single radiochemical species applying sys- tem 1 (2.1.2. Radiolabeling – Quality Control, in: Materials and Methods).

Preparation of [^{nat}Ga]Ga/[^{nat}In]In/[^{nat}Lu]Lu-DT14D

To an Eppendorf LoBind tube containing the DT14D stock solution (60 µL, 120 µg, ≈60 nmol, ≈1 mM), a solution (60 µL, 3 mM, 180 nmol) of the respective nitrate salt dis- solved in 1 M sodium acetate buffer of pH 4.0 (for gallium), 4.6 (for indium) and 5.0 (for lutetium) was added. The mixture was heated at 75 ^{0}C for 1 h. Complete binding of the metal by DT14D was demonstrated by RP-HPLC analysis of the reaction mixture using HPLC system 1-A, allowing for base line separation of [^{nat}In]In-DT14D and [^{nat}Lu]Lu- DT14D from metal-free DT14D. In the case of [^{nat}Ga]Ga-DT14D, base line separation from the metal-free peptide conjugate was possible only by ion-pairing using sodium 1-hep- tanesulfonate (10 mM in solvent A: 0.1% TFA in H_2O; solvent B: MeCN). Retention times of metal-tagged DT14D species are included in Table S2 (UV and gamma t_R were found identical in system 1-A). The metal-tagged DT14D solutions were used as such in the in vitro competition binding assays assuming a 0.5 mM NTS_1R-ligand concentration.

Table S2. Analytical HPLC data for (radio)metal-tagged TD14D (t_R (min) gamma/UV trace).

compound	DT14D	[$^{67/nat}$Ga]Ga-DT14D [a]	[$^{111/nat}$In]In-DT14D	[$^{177/nat}$Lu]Lu-DT14D
t_R (min)	18.8 / 22.1 [a]	21.2 [a]	20.3	20.2

A Symmetry Shield RP-18 (5 μm, 3.9 mm × 20 mm) cartridge column (Waters, Vienna, Austria) was eluted at a 1 mL/min flow rate with the following linear gradient (system 1-A): from 100%A/0%B to 80% A/20% B in 5 min and to 72.5%A/27.5%B in 5 min in 30 min; A = 0.01% TFA and B = MeCN; [a] A = 10 mM sodium 1-heptanosulfonate in 0.01% TFA – twin gamma and UV trace at 220 nm.

Biodistribution of [^{67}Ga]Ga/[^{111}In]In/[^{177}Lu]Lu-DT14D in Mice Bearing AsPC-1 Tumors

Biodistribution results for DT14D labeled with Ga-67, In-111 and Lu-177 in SCID mice bearing AsPC-1 xenografts, expressed as %IA/g and representing average values ± SD, n= 4, per animal group, are included in the following Tables S3-S5.

Table S3-a. Biodistribution of [^{67}Ga]Ga-DT14D in SCID mice bearing AsPC-1 xenografts at 1 and 4 h pi (NTS$_1$R-block and controls); data is expressed as average %IA/g values ± sd, n = 4 (for block n = 3).

Organs/Tissues	1 h pi	4 h pi	4 h pi – block [1]
Blood	9.52 ± 0.76	2.15 ± 0.40	1.86 ± 0.26
Liver	3.74 ± 0.37	3.44 ± 0.15	4.48 ± 0.86
Heart	2.86 ± 0.16	0.76 ± 0.12	0.77 ± 0.04
Kidneys	7.10 ± 1.12	3.87 ± 0.96	3.52 ± 0.66
Stomach	0.97 ± 0.27	0.45 ± 0.08	0.45 ± 0.02
Intestines	2.81 ± 0.53	2.32 ± 0.39	1.54 ± 0.16
Spleen	2.86 ± 0.33	1.75 ± 0.67	5.26 ± 1.54
Muscle	1.14 ± 0.15	0.27 ± 0.04	0.25 ± 0.02
Lungs	4.31 ± 0.28	1.34 ± 0.20	3.98 ± 1.16
Pancreas	1.41 ± 0.12	0.51 ± 0.08	0.49 ± 0.08
Femur	2.24 ± 0.23	1.67 ± 0.36	1.34 ± 0.16
AsPC-1 Tumor	11.42 ± 1.25	4.39 ± 0.62	1.88 ± 0.62

[1] Mice co-injected with excess NT (100 μg for NTS$_1$R blockade).

Table S3-b. Tumor-to-tissue ratios of [^{67}Ga]Ga-DT14D biodistribution in SCID mice bearing AsPC-1 xenografts at 1 and 4 h pi based on Table S3-a.

Tumor/Tissue	1 h pi	4 h pi
Blood	1.20± 0.06	2.06 ± 0.21
Liver	3.05± 0.19	1.28 ± 0.24
Heart	3.99 ± 0.24	5.75 ± 0.17
Kidneys	1.62 ± 0.13	1.15 ± 0.12
Stomach	12.20 ± 2.71	9.96 ± 1.68
Intestines	4.12 ± 0.52	1.89 ± 0.06
Spleen	4.00 ± 0.08	2.50 ± 0.23
Muscle	10.05 ± 1.09	16.50 ± 2.36
Lungs	2.64 ± 0.15	3.28 ± 0.21
Pancreas	8.09 ± 0.24	8.69 ± 0.52
Femur	5.10 ± 0.34	2.65 ± 0.25
AsPC-1 Tumor	1.00 ± 0.00	1.00 ± 0.00

Table S4. Biodistribution of [^{111}In]In-DT14D in SCID mice bearing AsPC-1 xenografts at 4 h pi (NTS$_1$R-block, controls, and Entresto® treated) and 24 h pi(controls and Entresto® treated); data is expressed as average %IA/g values ± sd, n = 4 (for block n = 3).

Organs/Tissues	4 h pi			24 h pi	
	block [1]	controls	Entresto® [2]	controls	Entresto® [2]
Blood	1.40 ± 0.71	0.66 ± 0.19	2.09 ± 0.18	0.46 ± 0.08	0.54 ± 0.18
Liver	0.76 ± 0.44	0.45 ± 0.04	0.90 ± 0.12	0.33 ± 0.08	0.34 ± 0.05
Heart	0.61 ± 0.34	0.28 ± 0.06	0.73 ± 0.07	0.62 ± 0.23	0.58 ± 0.14
Kidneys	6.15 ± 2.35	3.90 ± 0.20	6.78 ± 1.33	1.70 ± 0.13	2.34 ± 0.43
Stomach	0.43 ± 0.17	0.39 ± 0.05	0.54 ± 0.11	0.27 ± 0.10	0.28 ± 0.05

Intestines	1.27 ± 0.54	1.34 ± 0.06	2.22 ± 0.07	0.69 ± 0.09	1.04 ± 0.16
Spleen	1.44 ± 0.65	0.84 ± 0.07	1.44 ± 0.05	1.59 ± 0.14	1.77 ± 0.13
Muscle	0.24 ± 0.10	0.13 ± 0.03	0.37 ± 0.07	0.40 ± 0.10	0.43 ± 0.18
Lungs	1.75 ± 1.07	0.79 ± 0.12	1.99 ± 0.34	0.61 ± 0.09	0.88 ± 0.38
Pancreas	0.42 ± 0.18	0.24 ± 0.04	0.57 ± 0.07	0.63 ± 0.39	0.85± 0.69
Femur	0.76 ± 0.32	0.50 ± 0.06	1.10 ± 0.05	1.35 ± 0.36	1.20 ± 0.25
AsPC-1 Tumor	2.42 ± 0.23	3.73 ± 0.45	8.44 ± 1.06	2.82 ± 0.68	3.25 ± 0.11

[1] Mice co-injected with excess NT (100 µg for NTS$_1$R blockade). [2] Mice pre-treated with Entresto® (200 µL, 12 mg received per os 30 min in advance) as a sacubitrilat source.

Table S4-b. Tumor-to-tissue ratios of [^{111}In]In-DT14D biodistribution in SCID mice bearing AsPC-1 xenografts at 4 h (controls and Entresto® treated) and 4 h pi (controls and Entresto® treated) based on Table S4-a.

**Tumor/Tissue**	**4 h pi**		**24 h pi**	
	**controls**	**Entresto®**	**controls**	**Entresto®**
Blood	5.93 ± 1.64	4.04 ± 0.48	6.09 ± 0.82	6.54 ± 2.30
Liver	8.22 ± 1.14	9.47 ± 1.30	8.86 ± 2.85	9.74 ± 1.39
Heart	13.49 ± 3.44	11.50 ± 0.73	5.01 ± 2.02	5.97 ± 1.67
Kidneys	0.95 ± 0.08	1.30 ± 0.41	1.66 ± 0.40	1.42 ± 0.25
Stomach	9.56 ± 0.47	16.38 ± 5.27	11.28 ± 3.32	11.72 ± 1.63
Intestines	2.78 ± 0.34	3.80 ± 0.53	4.07 ± 0.42	3.16 ± 0.49
Spleen	4.44 ± 0.61	5.87 ± 0.75	1.77 ± 0.32	1.84 ± 0.15
Muscle	28.22 ± 5.43	23.09 ± 3.79	7.44 ± 2.40	8.45 ± 3.08
Lungs	4.84 ± 1.10	4.27 ± 0.45	4.55 ± 0.54	4.11 ± 1.31
Pancreas	15.66 ± 3.09	14.80 ± 0.84	5.47 ± 2.49	5.33± 2.48
Femur	7.53 ± 0.96	7.66 ± 0.64	2.11 ± 0.34	2.81 ± 0.69
AsPC-1 Tumor	1.00 ± 0.00	1.00 ± 0.00	1.00 ± 0.00	1.00 ± 0.00

Table S5-a. Biodistribution of [^{177}Lu]Lu-DT14D in SCID mice bearing AsPC-1 xenografts at 4 h pi (NTS$_1$R-block, controls), 24, 48 and 72 h pi; data is expressed as average %IA/g values ± sd, n = 4 (for block n = 3).

**Organs/Tissues**	**block [1]**	**4 h pi**	**24 h pi**	**48 h pi**	**72 h pi**
Blood	0.74 ± 0.11	0.72 ± 0.19	0.05 ± 0.01	0.05 ± 0.01	0.04 ± 0.00
Liver	1.41 ± 0.17	1.34 ± 0.14	1.18 ± 0.18	0.96 ± 0.05	0.86 ± 0.10
Heart	0.39 ± 0.18	0.32 ± 0.09	0.07 ± 0.01	0.07 ± 0.01	0.06 ± 0.01
Kidneys	4.66 ± 0.75	3.24 ± 0.20	1.24 ± 0.20	0.88 ± 0.06	0.57 ± 0.15
Stomach	0.30 ± 0.25	0.33 ± 0.12	0.06 ± 0.01	0.07 ± 0.01	0.04 ± 0.01
Intestines	0.80 ± 0.14	1.27 ± 0.14	0.45 ± 0.03	0.36 ± 0.01	0.27 ± 0.02
Spleen	1.64 ± 0.29	1.36 ± 0.37	1.06 ± 0.16	1.60 ± 0.58	1.49 ± 0.51
Muscle	0.17 ± 0.07	0.11 ± 0.02	0.03 ± 0.00	0.04 ± 0.01	0.04 ± 0.02
Lungs	1.41 ± 0.38	1.63 ± 0.86	0.17 ± 0.01	0.14 ± 0.00	0.13 ± 0.04
Pancreas	0.28 ± 0.10	0.21 ± 0.03	0.08 ± 0.00	0.07 ± 0.01	0.08 ± 0.02
Femur	0.62 ± 0.15	0.45 ± 0.06	0.34 ± 0.03	0.38 ± 0.03	0.35 ± 0.03
AsPC-1 Tumor	2.01 ± 0.16	3.77 ± 0.59	1.77 ± 0.13	1.56 ± 0.17	1.24 ± 0.20

[1] Mice co-injected with excess NT (100 µg for NTS$_1$R blockade).

Table S5-b. Tumor-to-tissue ratios of [^{177}Lu]Lu-DT14D biodistribution in SCID mice bearing AsPC-1 xenografts at 4, 24, 48 and 72 h pi based on Table S5-a.

Tumor/Tissue	4 h pi	24 h pi	48 h pi	72 h pi
Blood	5.51± 1.56	33.51 ± 2.94	30.93 ± 3.67	29.76 ± 7.43
Liver	2.83 ± 0.45	1.54 ± 0.32	1.63 ± 0.14	1.44 ± 0.19
Heart	12.36 ± 3.08	25.00 ± 3.78	22.97 ± 2.32	20.05 ± 3.03
Kidneys	1.17 ± 0.15	1.46 ± 0.33	1.78 ± 0.30	2.26 ± 0.49
Stomach	12.29 ± 3.81	31.97 ± 8.07	23.81 ± 2.17	26.61 ± 5.58
Intestines	2.99 ± 0.57	3.95 ± 0.39	4.28 ± 0.33	4.65 ± 0.91
Spleen	2.83 ± 0.35	1.70 ± 0.32	1.10 ± 0.51	0.89 ± 0.25
Muscle	34.86 ± 6.98	58.07 ± 8.34	39.22 ± 5.49	36.07 ± 14.62
Lungs	2.97 ± 1.75	9.32 ± 2.56	11.41 ± 1.00	9.66 ± 1.43
Pancreas	18.16 ± 3.31	22.31 ± 1.64	20.41 ± 1.96	16.25± 5.04
Femur	8.43 ± 2.01	5.30 ± 1.64	4.14 ± 0.47	3.53± 0.33
AsPC-1 Tumor	1.00 ± 0.00	1.00 ± 0.00	1.00 ± 0.00	1.00 ± 0.00
